# Multiple Eschars in Scrub Typhus

**DOI:** 10.4269/ajtmh.23-0105

**Published:** 2023-07-03

**Authors:** Harpreet Singh, Suresh Selvam, Vikas Suri, Manisha Biswal, Ashish Bhalla

**Affiliations:** ^1^ Department of Internal Medicine, Post Graduate Institute of Medical Education and Research, Chandigarh, India;; ^2^ Medical Microbiology, Post Graduate Institute of Medical Education and Research, Chandigarh, India

**Figure 1.**
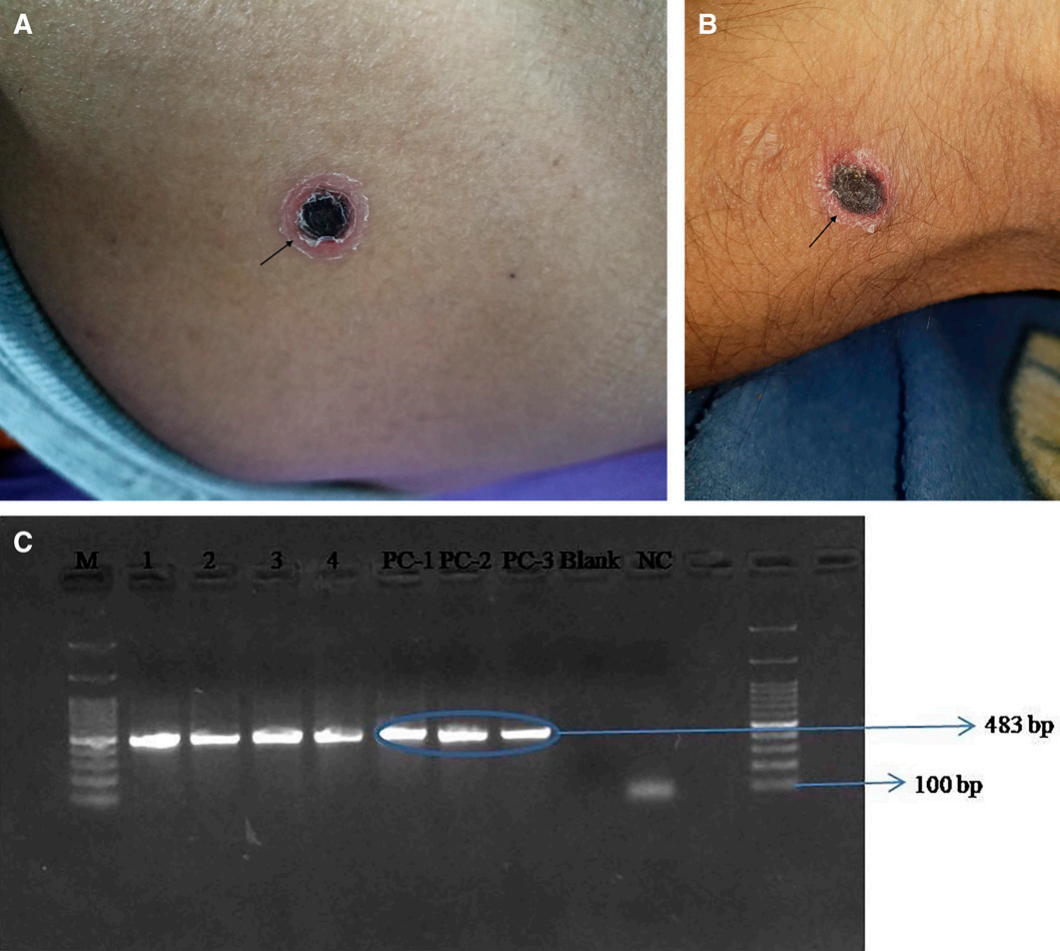
(**A**) Eschar on the left gluteal region with characteristic brownish black crust surrounded by annular red halo. (**B**) Eschar near the right popliteal region with characteristic brownish black crust surrounded by annular red halo. (**C**) Gel electrophoresis image of results of nested polymerase chain reaction for the detection of 56 kDa gene (partial) of *Orientia tsutsugamushi*.

**Case:** A 47-year-old male without any comorbidities was admitted with an intermittent fever of up to 102°F associated with chills and rigors and generalized weakness of 7 days duration. On examination, systolic blood pressure was 112 mm Hg, diastolic blood pressure was 68 mm Hg, pulse rate was 102 per minute, and respiratory rate was 18 per minute. Laboratory investigations showed hemoglobin of 9.9 g/dL, total leukocyte count of 12,600/mm^3^, and platelet count of 12,000/mm^3^, raised total bilirubin of 1.06 mg/dL, alanine aminotransferase of 50 U/L (2–41 U/L), aspartate aminotransferase of 90 U/L (2–40 U/L), and raised international normalized ratio of 1.48. The possibility of tropical infection like scrub typhus, leptospirosis, and malaria were considered because the patient had presented in the post-monsoon season. Physical examination revealed two eschars: one on the left gluteal region (Figure 1A) and the other near the right popliteal region (Figure 1B). Serum IgM ELISA (InBios International, Inc.) was performed. The cutoff optical density (OD) of this kit according to the manufacturer’s instructions is 0.468, and the OD of this patient’s sample was > 4.0 (positive). Nested polymerase chain reaction for the partial 56 kDa gene was determined for both eschars and was positive for *Orientia tsutsugamushi* (Figure 1C).^1^ The positive amplicons were sent for the Sanger sequencing. The sequences were submitted to NCBI with following accession numbers: OQ214881 and OQ214882. These sequences were found to be similar to previously reported *Orientiatsutsugamushi* isolates from Post Graduate Institute of Medical Education and Research, Chandigarh (OP651876, OP651822) with percent identity 100% and query coverage ≥ 83%. Both the sequences also showed > 99% identity and ≥ 99% coverage with *O. tsutsugamushi* isolate MM2018-99 genotype Kato-B reported from Myanmar. The patient was treated with doxycycline 100 mg twice daily for 7 days and discharged. Work-up for other tropical infections (leptospirosis, dengue, and malaria) was negative. There was no relapse of fever at 3-month follow-up.

Scrub typhus is one of the remerging rickettsial infections in Southeast Asia caused by *O. tsutsugamushi*, which is an intracellular gram-negative organism.^2^ There was a surge in cases from the endemic area during the 1930 s and later on in World War II, and there was fall in reported cases over two decades after the 1970 s. However, there has been an increase in the number of cases of scrub typhus since that period.^2^ Various factors linked to this re-emergence of infection include development of antimicrobial resistance to chloramphenicol and tetracyclines (used previously for common infections), ecological changes (deforestation and urbanization), and various climatic factors like global warming.^3^^,^^4^ An eschar denotes the site of bite of chiggers (the larval stage of trombiculid mites; *Leptotrombidiumdeliense*) with multiplication of *O. tsutsugamushi* at the local site. It is usually formed within 5 days of mite bite and may heal in several weeks. It usually starts as small vesicle or erythematous plaque followed by central ulcer of 0.5–3 cm covered with a brown-black crust. It is surrounded by an annular red halo and looks like a small depressed scar in healing stage.^5^ The presence of an eschar is highly suggestive of rickettsial infection.^5^ However, eschars are variably present in patients of scrub typhus (range: 7–97%), as reported in studies throughout the endemic regions.^6^ An absence of eschar may be due to differences in geographic distribution of various genotypes of *O. tsutsugamushi* or an insufficient physical examination.^6^ There are only few case reports of multiple eschars in patients with scrub typhus infection.^6^^–^^9^ Eschars are found predominantly over the chest and abdomen in females and over the axilla, groin, and genitalia in males.^9^ Eschars may contain abundant amount of rickettsial DNA, which is why polymerase chain reaction of eschar biopsies or vigorous swabs of eschar areas is helpful in providing definitive evidence of the disease in early stages before seroconversion. It can be hypothesized that chiggers bite the human host multiple times, leading to more than one eschar; however, there are no definitive epidemiological studies to prove this theory. Treatment with doxycycline should not be delayed in the absence of serological evidence if the clinical picture is suggestive of rickettsial infection with the presence of eschar.
